# Optimizing Care for Ketamine Use Disorder: An Interdisciplinary Treatment Model

**DOI:** 10.1192/bjo.2025.10396

**Published:** 2025-06-20

**Authors:** Rebecca McKnight, Ijeoma Chibuzo, Tracey Myton, Ling Lee

**Affiliations:** 1 Greater Manchester Mental Health NHS Foundation Trust, Manchester, United Kingdom; 2 Bolton NHS Foundation Trust, Bolton, United Kingdom

## Abstract

**Aims:** Ketamine use among young adults in England has increased significantly, with prevalence more than doubling in the past five years. Ketamine use disorder (KUD) is a disorder of regulation arising from repeated or continuous use of ketamine for at least three months. The systemic effects can include urinary, sexual, hepatic and cardiovascular dysfunction, memory impairment and mental illness. Although people who use ketamine constitute a smaller proportion of patients in addictions services compared with opioid or alcohol users, the complexity and morbidity of KUD dictates the need for interdisciplinary collaboration. In 2024, a collaborative effort between a local addiction and urology service was initiated to address KUD and ketamine uropathy (KU).

**Methods:** Both services presented at the local Addictions Continuing Professional Development Day to share knowledge and develop staff understanding on KUD and KU. Meetings were held to evaluate local prevalence of KUD and KU, address barriers to treatment and develop easier referral pathways into both services. Best practice guidance on KU was reviewed and a new interdisciplinary treatment model implemented. Re-strategisation required clinician time and adjustments to clinic schedules.

**Results:** In 2024, nine patients from Urology and 23 patients from the addiction service with ketamine use were seen. Key improvements included the establishment of a direct two-week referral pathway to Urology, development of referral and assessment proformas and initiation of monthly interdisciplinary team meetings. These changes aimed to reduce delays in initiation of treatment and improve co-ordination between services. However, the major challenge faced was a high attrition rate in the clinics.

Areas identified as requiring further attention included management of weight loss and constipation, medication for symptomatic relief of ketamine withdrawal and cravings, safe analgesic alternatives, treatment of co-occurring mental illness and trauma, safeguarding and risk considerations and psychological therapeutic options. The embarrassment of urinary incontinence was identified as a barrier to appointment attendance.

**Conclusion:** An interdisciplinary management approach is recommended to optimize patient care. Systemic complications of KUD and co-occurring mental illness should be treated simultaneously. Intensifying the support from Addiction Recovery Coordinators may improve attendance at appointments.

Recommendations include more health worker education and staffing, early pain team involvement and provision of harm reduction advice. Peer-informed, ketamine-focused psychosocial programmes and national psychiatry guidelines for KUD are required.

Our collaborative model demonstrates a significant step towards improving management of KU and KUD, however its impact on clinical outcomes will need further evaluation.

